# Traumatic Pericarditis of the Ox1Read at the December meeting of the Missouri Valley Veterinary Medical Association.

**Published:** 1898-03

**Authors:** R. C. Moore

**Affiliations:** Kansas City, Mo.


### TRAUMATIC PERICARDITIS OF THE OX.[1]

By R. C. Moore, D.V.S.,
KANSAS CITY, MO.

In presenting this subject to the Association I desire to give a short history of two cases that came under my observation during this year, as a basis for discussion of this common accident and its differential diagnosis.

Case I.—The subject was a shorthorn milch-cow, belonging to Mr. Quistgart, living a few miles east of Kansas City, Mo. She was a large, eight-year-old cow, in good condition, weighing about 1200 pounds. First seen January 5, 1897. Had been well up to the previous day, when she refused to eat and showed signs of pain. Respiration slightly increased and painful, grunting with each inspiration. Temperature 103° F.; pulse accelerated, small, and hard. Appetite lost and rumination suspended, with some tympanitis. Alvine discharge wanting. Auscultation and percussion of the thorax revealed nothing abnormal except diminishing heart-sounds. Diagnosis: Indigestion, with possibly inflammation of the omasum. Prescribed saline cathartics with nux vomica. This was the last I saw of the cow alive, but her condition was reported to me two or three times a week by the owner, until she died three weeks later.

During the first eight to ten days the bowels remained inactive, in spite of the repetition of the previous medication. After the tenth or twelfth day an offensive diarrhœa was present, lasting to the end. Not seeing the patient after the first visit, I am not able to report any of the cardiac symptoms. Later the owner was advised of the probability of a cardiac traumatism.

Post-mortem revealed the pericardium greatly distended with a purulent fluid, amounting to, at least, two gallons. The pericardium was greatly thickened and firmly adhered to the diaphragm, and its corresponding inner surface adhered to the apex of the heart.

The epicardium was also thickened, deeply furrowed, and of a grayish-white color. A piece of bailing-wire, five inches long, was found in the contents of the sac, with well-marked traces of its previous penetration of the walls of the reticulum, diaphragm, pericardium, and heart, the end having penetrated the wall of the left ventricle to some depth. No other lesions were found.

[1] Read at the December meeting of the Missouri Valley Veterinary Medical Association.

Case II.—A Holstein cow, seven or eight years old. This case came under my care through a brother practitioner being called away from the city, leaving his patients with me. From history obtained from the owner, concerning the cow, she had taken sick about two weeks previously with symptoms similar to those reported in Case I., and had received about the same course of treatment.

July 3d, when I first saw the cow, I found the temperature 103° F.; hurried but not painful respirations; pulse accelerated, small, and feeble. Auscultation showed respiratory murmur wanting in the lower half of the left side of the thorax, and nearly as bad on the right side. The same area gave a dull sound on percussion. In the upper part of the chest the respiratory murmur was clear, but louder than normal. No heart-sounds were audible, but a clear, tinkling sound could sometimes be heard in the cardiac region. A cough was also present, as well as a dropsical swelling beneath the sternum, extending upward nearly to the throat. Deglutition was difficult; the cow was very weak, and rapidly losing strength from an exhaustive diarrhœa. Diagnosis: Hydrothorax. Prescribed stimulants and tonics with iodide of potash. Saw the cow every two or three days from then until she died, some ten days later.

Symptoms remained the same until two days prior to death, when an extremely offensive odor was present. Neither the breath nor feces gave this odor to any marked degree, yet the stable seemed to be permeated with it.

Dr. S. Stewart assisted in the post-mortem, which revealed the pericardium greatly distended, nearly filling the inferior half of the thoracic cavity. The contents, a very offensive, purulent liquid of a brownish color, not measured, must have been more than three gallons. Lying loose in this liquid we found a piece of an umbrella-stay (the stay that supports the ribs on the handle) about six inches long. No other lesions were noted.

These foreign bodies usually, if not always, enter the pericardium from the reticulum, the anatomical arrangement of the inside of which is well adapted for entangling any indigestible substance, the honeycomb or pockets of which can easily entangle a pointed substance, and should it become directed toward the diaphragm, the pressure from the rumen behind acting with the contractions of the diaphragm, tend to force the sharpened end through its walls. The pulsations of the heart assist in forcing it through the pericardium as well as the walls of the heart itself. Once a foreign substance has entered the pericardium, even though it is aseptic, it sets up sufficient irritation to cause inflammation with an outpouring of serum. Should the foreign body be septic purulency results.

Many cases of cardiac traumatisms are recorded. Gamgee reports one observer having found a table-knife, seven and one-half inches long, that passed from the reticulum to the left ventricle, and in another case a ramrod, fourteen inches long; also a pomegranate thorn was found under one of the left auriculo-ventricular valves, with a fistulous tract extending to the reticulum.

Some authors think needles and other small, sharp bodies pass from the œsophagus to the pericardium; but in most instances evidences of them having passed from the reticulum are marked. All observers agree that many foreign substances, even to pieces of bottles, are often found in the reticulum of the cow. Especially is this true of cows that are allowed to run at will about town; in fact, so fond are they of chewing such things that but little rubbish is exempt from their efforts to devour.

In this class of ailments of meat-producing animals where successful treatment is beyond our reach, a positive, early diagnosis is to be desired, for in the early stages the flesh might be used for food, which otherwise would be lost to the owner.

The earlier symptoms are, as a rule, associated with disturbances of the digestive tract, and, as derangement of the latter greatly predominates, it is but natural that the cardiac lesions be overlooked and valuable time lost. We find a great variety of symptoms of catarrhal affections of the stomach and bowels. Digestion will, as a rule, be suddenly suspended. As a result of this, these organs are distended with gas, peristalsis is arrested, and constipation follows, all as a result of shock to the system from the wound to the heart, or the irritation to the heart produces an increased peristalsis, and there is an increased and frequent alvine discharge. Temperature rarely rises more than one or two degrees.

These symptoms being so common in cattle-practice, due to other causes than cardiac traumatism, in which the practitioner would usually give a favorable prognosis, makes the cardiac symptoms very valuable, especially in animals known to be so liable to this lesion.

Symptoms furnished by the heart: Here again we find a variety of modifications. The pulse may be accelerated, wiry, hard, and small, or it may be extremely feeble. In either case the modifications will greatly exceed the disturbances of the digestive organs or body-temperature. The heart-sounds would not be greatly changed except they be feeble and less distinct than normal. Percussion sometimes reveals an increased resonant area over the heart.

The serious nature of the cardiac symptoms lead us to think of the heart being the member primarily disturbed.

As idiopathic lesions of the heart are rare in otherwise healthy animals, we would be at least reasonably safe in suspecting a traumatism, especially if the subject be a cow that is allowed to roam about town, where she has access to all kinds of rubbish. When an exudate fills the pericardial sac the pressure compresses the auricles and causes a passive hyperemia of the pulmonary tissue, causing cough, dyspnœa, and increased respiratory movements. Pneumonic symptoms are then likely to predominate and obscure the cardiac lesion, but the disturbances of the pulse and persistence of the vesicular murmur should prevent error.

As a further result of the exudate pressing on the venous trunks the jugulars become engorged and distended, causing a venous pulse. There may be splashing of water with the pulsations of the heart, or a tinkling, metallic sound from pericardial tension. But the blowing and whistling sounds common in valvular insufficiency are not to be expected.

The obstructed venous circulation will be likely to produce anasarca of the inferior parts of the body as well as dropsy of the cavities, but these are not diagnostic symptoms.

The duration of the disease is variable. If the heart is not seriously wounded, life is often prolonged for weeks or even months, and death is due to exhaustion, or complication resulting from a weakened circulation, or as a result of pyemia or septicemia from the purulent effusion.

Should the heart be seriously wounded death might result at any moment after the wound is inflicted.

A few cases of recovery have been reported by retrogression of the foreign body into the stomach or its destruction by chemical process.

---

AMONG the veterinarians interested in the bottled-milk question at the several meetings of the Sanitary Committee of the Board of Health of Philadelphia were to be noted Drs. Leonard Pearson, M. E. Conard, W. L. Nunan, W. Horace Hoskins, E. B. Ackerman, of Brooklyn, B. F. Senseman, Frank L. Smith, Charles Earnest, G. R. Hartman, with many prominent physicians and bacteriologists.

